# On the Effects of the Eau Medicinale, of Elaterium, and of Purgatives, in the Cure of Gout

**Published:** 1814-11

**Authors:** Tho. Sutton

**Affiliations:** Croom's Hill, Greenwich


*For the Medical and Physical Journal.*

### *On the Effects of the Eau Medicinale, of Elaterium, and of Purgatives, in the Cure of Gout;* by T. SUTTON, M.D.

I PERCEIVE my letter on Mr. Want's discovery of the composition of the Eau Medicinale, has been inserted by him in the Medical and Physical Journal, for the purpose of making his remarks on it. He appears to be much displeased that I have maintained, so far as he can comprehend, the infallibility of purgatives, in the cure of gout, through the whole paper; but is not surprised to find that I consider them to be as efficacious in this disease as his favourite medicine, because I either am, or appear to be, ignorant of the virtues of the latter. Mr. Want might, however, have readily saved himself the trouble of asserting, that I was "totally unacquainted with that curative property of the eau medicinale to which he alludes, or the sensible effects of that remedy," by adverting to one part of my paper, which he could not, I presume, have readily overlooked, where I have stated, that "these (advantages of the French medicine) appeared to me to be effected in the most material and permanent degree by a powerful action on the bowels, although it certainly more immediately allays the violence of pain by its anodyne quality."

My Tract on Gout expressly points out three ways of subduing a paroxysm of this disease,—by purging, by the use of anodynes, and by cold applied to the part affected. I have considered the cure by the use of purgatives to be the most perfect, combined either with anodynes, or by giving an anodyne after their operation. The latter mode of proceeding is exemplified by the case related in my Tract on Gout, page 202; and I also judged that the operation of the eau medicinale in the cure of the gout, was most efficient in those cases where it acted on the bowels. I always thought the beneficial effects of this medicine in gout to consist in an anodyne, or soothing, and purgative quality, and therefore recommended elaterium and opium to imitate them to this extent. I could not be insensible of the powers of an anodyne dyne to subdue a fit of the gout, for in page 213 I have given a case of the most complete submission of the paroxysm of this disease to the use of laudanum; and in page 267 I have stated that similar effects resulted from the use of opium. I had not, therefore, overlooked Mr. Want's favourite quality of the eau medicinale exactly to the extent with which he was willing to impress his readers, nor was I ignorant of the analogous and beneficial effects of other medicines in imitation of this quality. This supposed want of information, therefore, on my part, not being so very complete as he has stated, I do not find that Mr. Want has written any thing to invalidate my intimation, that the eau medicinale possesses "no greater (beneficial) efficacy in curing the gout than many known remedies," and "that we possess a numerous class of medicines which are equally as capable of subduing the paroxysms of gout as the eau medicinale."

Mr. Want states, that the beneficial properties of the eau medicinale, or of the colchicum autumnale, for the cure of gout, are quite distinct, from its purgative powers. That he, however, should refer all the good in the gout to these properties alone, is rather singular, when he admits that purgatives exercise a beneficial influence on the disease, although he infers, that they fail oftener than the eau medicinale or his preparation. The soothing powers, however, of the eau medicinale, of opium, and of cold applied to the parts affected, are in this respect analogous in effect, that they suspend the paroxysms; but I contend, that they do not exercise over the disease so powerful and effectual an ascendancy as to reduce the cause of gout to a long subjugation of its influence on the system, or entirely to overcome it. The former of these, the use of purgatives, properly administered, with attention to some few other circumstances, will, in most cases, effect; and the latter appears to me to be often attainable.

I have taken this opportunity to examine the facts that inclined me to consider the eau medicinale to be a purgative. I find, from Dr. Jones's account, that Huson recommended it "from a view to its evacuating powers;" (vide Jones on the Eau Medicinale d'Huson, page 6th;) and, from his own observation (Dr. Jones's) "that sometimes it produces no evacuation at all; at others, it proves powerfully emetic and cathartic." Out of twenty patients mentioned by Dr. Jones, who took the French medicine, four were not purged, three very moderately, and eleven powerfully. In Mr. Stanhope's case, it remains doubtful whether the bowels were affected or not; and in one there is no notice taken of the operation of the medicine in any other way than that it relieved the paroxysm of gout. Among those on which the eau medicinale cinale acted powerfully, is included the case of Sir Joseph Banks. Dr. Jones states, "forty-eight hours after Sir Joseph had taken the first dose, the remaining part of the bottle was administered, and, in the course of the next twelve hours, the medicine began to operate; and procured five evacuations." Mr. Want says, "Sir Joseph assures me, that in him the eau medicinale never produces any action on the bowels, while it never fails to relieve him."

Mr. Want, I find, in several instances, has made use of italics for the purpose of drawing the attention of his readers to several points in his assertions which have a reference to me, and which he has not omitted to do on the subject of his claim to the credit of first using powerful purgatives, especially elaterium, for the cure of the paroxysms of gout. He has, however, done me the favour to state, that he first employed these medicines in the year 1811, which will readily give me an opportunity of deciding the question between him and me on this subject. Whether the purgative effects of the eau medicinale, or those which tend to lull the pain or keep in check the paroxysm of gout, shall be considered to be the properties in which the chief virtues of this medicine reside for the cure of the disease, it is extremely evident, that Dr. Jones's book affords, at least, ten cases in which it caused a powerful action on the intestines. And, if we admit Mr. Want's opinion, which it is not necessary here to contend about, it must be evident, at least negatively, in reading these cases, that the operation of powerful purging was not attended with detriment. My experience of the efficacy of cathartics in gout, to the extent to which I had then employed them, led me to conclude that the purgative effects of the eau medicinale were desirable and beneficial in that disease. It was with these impressions I first advanced to the recommendation of a powerful cathartic action on the bowels in the paroxysms of this disorder; and I can safely affirm that I had used medicines to produce this effect previous to June 1810, and that I had communicated my opinion of their efficacy to some medical friends, and at a meeting of a society called the Kent Medical Society, which is composed of medical men from town or its vicinity; that I communicated the prescription of elaterium and opium for the cure of gout to many of the members, either at the May or June meeting, 1810. In June of the same year, I attended a patient with Dr. Heberden at Chesilhurst, where the effects of the eau medicinale on gout was the subject of conversation. Dr. Heberden there said he had seen Huson's book, and I mentioned to him my preparation as a substitute for the eau medicinale, wrote it down, and gave it to him.

 The

The formula was two grains of elaterium, and sixty drops of tincture of opium, in an infusion of ginger. In Sept. 1810, I was attacked with gout, and employed purgatives with the most marked success. I transmitted my case to Dr. Lettsom for the Medical Society in Fleet-street, of which he was president; and it was read at some of its meetings before Christmas 1810, as Dr. Lettsom informed me by letter. In the beginning of 1811 I transmitted another letter to Dr. Lettsom for the Society, communicating two cases of the efficacy of elaterium in the cure of gout, by Mr. Green of Lewisham; whether these were read or not, I cannot vouch. From this statement, it must be evident, that I had communicated my opinions of the advantages of elaterium and other purgatives, through different channels, before Mr. Want ever thought of using them. I cannot say whether he afterwards employed them, through suggestions arising in his own mind, or in consequence of any hints he might have received, and which he has forgotten. But, in so far as the priority of claims to the use of these medicines exists, between Mr. Want and myself, I believe I have said sufficient to decide the question.

I do not pretend to account for Mr. Want's failures in the use of cathartics for the cure of gout; I have only related such results as have occurred to me during four years' practice in the use of these medicines; and Mr. Want's communications hitherto on the subject of the eau medicinale, and of his composition as a substitute for it, have only given me a more lively and satisfactory conviction, that I have performed a most essential service to the public in recommending a cure for gout chiefly grounded on the operation of purgatives, and I doubt not that it will survive all the different speculations on the cure of that disease, or the various remedies which have been of late proposed, not excepting the eau medicinale or the colchicum autumnale.

If we were only to rely on the eau medicinale, or Mr. Want's preparation, many cases of gout would occur which must be left unaided, because it would be next to madness to think of administering such compositions in them. I have had occasion frequently to see a lady who has for years been a great martyr to the gout, and who is never attacked with this disease without its being attended with great sickness and costiveness. Previous to her taking cathartic medicines, paroxysms of gout so accompanied have continued with her for a considerable time, and she has been confined to her bed, or her room, when so affected, for six weeks and upwards. This state of disease is now soon dissipated, and the gout vanishes, as soon as the bowels can be got to act in a relaxed manner, manner, which is generally effected in two or three days, should there be room for the exhibition of the eau medicinale in such a case as this, yet the disease soon yields to the use of purgatives.

A gentleman who had laboured under several severe and tedious fits of the gout, was attacked with hemiplegia. At the time I am speaking of, this was the second attack of paralysis. The disease now indicated much determination to the head, and was accompanied by a great loss of power on the side affected, as well as of a considerable affection of the speech. About ten days after this attack, at which time the patient had shewn some signs of the probability of recovering the use of his limbs to a considerable extent, a fit of the gout came on. It must be evident to every one, if this disease continued so long as it had usually done in former attacks, that the progress in amendment, in so far as it respected the paralytic affection, must be much retarded; and it was also probable, by an inactivity of some duration, that the period might pass over in which so desirable an object could to any extent be accomplished. Under these circumstances, as I could place much confidence in the powers of purgatives over the gout, I recommended the attending surgeon to administer a powerful medicine of that sort: it produced an appropriate effect, and the gout was subdued. From this time, eighteen months ago, the patient has enjoyed a freedom from this disease, and has regained the use of his limbs to full as great an extent as could, at any time, have been anticipated. This also, I presume, may be ranked as a case in which the eau medicinale could not with propriety have been advised. No other medicines could have been eligible for the cure of gout in these two cases, it is therefore I have related them; but there are many others which can be overcome by the use of this class of medicines, sometimes with the occasional aid of opium, and at other times without it, in which such a medicine as the eau medicinale could not be thought of.

In all my trials with the use of purgatives for the cure of gout, I have the satisfaction to know, that not one circumstance has occurred to throw a suspicion on the propriety of the practice, in regard either to the immediate or ulterior state of the patient, nor of dissatisfaction respecting its results, excepting the case before remarked. In regard to the eau medicinale, it has happened otherwise. Several of those with whom I have conversed, have grown tired of the medicine, because it has afforded them only a short reprieve, without a thorough restoration of healthy feelings, and therefore they have determined to submit to a severe fit of the gout, to be able, even for a short time, to attain to a more perfect perfect state of health. From medical men, also from many quarters, I hear of patients who have taken the French medicine, whose health and appetite have been impaired, and who have not only lost all confidence in the eau medicinale, but who have regretted exceedingly their ever having employed it. Dr. Gregory, of Edinburgh, believes he has had one patient who died in consequence of taking this medicine, by producing a violent action on the bowels, and another who was attacked with water in the chest two successive times, after he had taken the same medicine. In two cases after the use of this nostrum, I have seen obstinate constipation to ensue, in one of which the disease ended in death, and in the other case, the bowels could not be brought to act for several days. Mr. Ring and Dr. Adams have each of them, I believe, seen a fatal case from the use of this composition.

With what different sensations must a practitioner prescribe such remedies as purgatives for the cure of gout, which when exhibited in this disease, unless very much mismanaged, can produce no untoward symptom; and when he approaches the bed-side of a patient with a medicine which, granting Mr. Want's preparation and the eau medicinale to be the same, is, according to him, "endued with properties most unmanageable and deleterious," and which, in one instance, in spite of all Mr. Want's address, nearly cost the patient his life. "He has (also) seen cases in which it produces a most alarming sense of suffocation, from the globus hystericus, and flatulent distension of the abdomen." When we go on farther with this comparison, we shall find, after this deleterious drug has been administered, that "out of forty patients, in several instances no return of the disease has taken place for several months." Among those who have confided in purgatives, however, for the cure of gout, I can adduce those who have enjoyed health and freedom from disease for a much longer period, among whom I have the happiness to include myself, who have now been free from the gout for upwards of four years.

As this paper has now extended to a considerable length, I shall not dwell on the least important part of Mr. Want's controversy with me, whether he has discovered the composition of the eau medicinale or not. I, however, hope he has, and I have so far changed my opinion under that impression, as to hope also that the discovery will be of considerable advantage to the public, by an inverse proceeding, however, to that which Mr. Want has aimed at. It can now, I apprehend, no longer be contended, that the knowledge of the composition of a nostrum is of none or very little consequence to those who recommend it, since, admitting Mr. Want's discovery, we find that the public has been presented, in the form of the French medicine, with an unmanageable and pernicious drug, which, although a solitary or unfrequent use of it, in a moderate dose, may not draw down most serious and baneful effects upon the system in every case, because of the admirable powers which are connected with our existence to resist to a certain extent things which operate to its destruction, yet it cannot be supposed that the frequent use of this deleterious plant can ever act in concurrence with the entire and perfect health of the human frame. If there are paroxysms of gout which this medicine can controul better than any other, which I doubt, let it be given to that extent, and then let the powers of purgatives complete the work of restoration. In this limited way such medicines may be admissible, and in this way also the eau medicinale or Mr. Want's composition will leave less deleterious effects on the system. For, whatever may be maintained to the contrary, it must appear evident, that it would be desirable to throw such a medicine out of the habit, so soon as can be done after it has effected its beneficial purposes, and that the purging powers of this drug are adapted, if to effect no other good, to ward off the unhappy consequences that might ensue from its continuance in the habit. Mr. Want has even admitted that some of the alarming effects of this medicine are prevented, when it takes to operate on the bowels, for, after enumerating some serious symptoms brought on by the colchicum autumnale, when it does not act by purging, he adds, "though most commonly in a full dose it has this effect (purging), which, if it does take place, immediately removes the sense of suffocation."

It was the unlimited recommendation of this medicine to the public, and in a popular publication, which induced me to notice Mr. Want's supposed discovery as I have done; while at the same time he avoided giving any intimation of the benefits of a class of medicines of great efficacy and powers, in controuling and in curing the gout; and, however Mr. Want may have failed in their use, he cannot help mentioning them with commendation. But let it be remarked, that the tendency of his first paper on this subject had in view to recommend exclusively his composition; for, in the Monthly Magazine, he introduces his discovery, by announcing it to be a "very important preparation;" he recommends its use on account of its cheapness to persons in the labouring class of the community, who on account of the high price of the eau medicinale were not able to obtain it; and he hints that the unwillingness of the profession to introduce the French nostrum, nostrum, has been owing to its being a secret medicine, which will be removed by his discovery; and, lastly, although he acknowledges that his composition is unmanageable and deleterious, he states that "he has used it, at least in forty cases, with the most satisfactory results." If my notice of Mr. Want's paper will be followed by no other advantages, it will make more extensively known the use and benefits of purgatives in gout, and acquaint the public, that the gouty may have a chance of losing the disease, without having recourse to a medicine that may bring with it more serious evils than it is capable of driving away. I however find that Mr. Want has been more limited in recommendation of his preparation, in his reply to me. For in that he says, "I simply state that this medicine does that which the eau medicinale is capable of performing, and I recommend it to those only who have experienced the good effects of that remedy." If Mr. Want had held out no other inducement beyond the last for its use, I should certainly not have been at the pains of taking notice of his discovery, nor should I have made any observations at all on his communication, if it had appeared in a Medical Journal as announced and published in the Monthly Magazine. I judged it to have a direct tendency to fix the public mind to the exclusive use of a drug for the cure of gout, which its admirers cannot consider, in many points of view, with commendation.

Lastly, as Mr. Want is become the legitimate defender of the eau medicinale, and of his discovery, though only, as I presume, with a view to public usefulness, I would recommend him to solicit from all quarters communications to be addressed to him, on the effects of the French medicine and his own, in so far as they may have appeared to be detrimental, either on their immediate use, or at a subsequent period. Such information will, I should hope, have a tendency to fix the public mind, as to the propriety of confiding in, or giving countenance to, medicines possessing noxious qualities, for the cure of the gout; while their superior efficacy, if any, in their quicker operation, and the certainty of the relief they afford, does not much exceed a mode of cure perfectly safe in itself, and which also possesses the advantages of a more extensive application, connected with the probability of more permanent benefits.

*Croom's Hill, Greenwich,* THO. SUTTON.

*Sept.* 10, 1814.

P. S.—*October* 1.—I perceive Mr. Want has stated as follows in a communication in the Medical and Physical Journal of the present month: "It was observed by Dr. Sutton,

 that that the eau medicinale produced its beneficial effects by its purgative operation; and distinctly asserts, that even common purgatives are capable of removing the disease with equal facility. For the complete refutation of both these assertions, I (Mr. Want) may refer to our last Number, where the subject is considered at length." Mr. Want has no more completely proved, I beg to assert, that common purgatives are not capable of removing a fit of the gout, than he has proved that he employed purgatives and elaterium before me. Both rest upon his assertion: the latter I have completely refuted, but cannot do so with the former, otherwise than by referring to my communications and tract on Gout. This then must depend on the relative credit we enjoy with the public.

---

*Letter addressed to* Dr. Sutton.

Sir,—On perusing Mr. Want's answer to your remarks upon the preparation of a medicine which he conceives to be the true eau medicinale, he says, he is perfectly convinced, from the united testimony of several distinguished literary characters, that the two compositions are identically the same. Mr. Want needed not their testimony, if he had acknowledged the having obtained a specimen of the plant* from which the French remedy was prepared, (this Mr. W. stated at a meeting of the Westminster Medical Society, in Windmill-street, during the last winter,) it having been given him by the housekeeper of the preparer or vender of that medicine, during his attendance upon her. This, I think, is *conclusive*, as to the identity of the two remedies. Before Mr. W. is allowed the discovery of the remedy, it remains with him to prove his use of it prior to his attendance on the person alluded to. Being unwilling to enter into any discussion, I merely send you this fact, well known to the members who were present upon that occasion, to use in any way you think proper.

*Sept.* 9, 1814. *An occasional Visitor of the Society.*

---

FROM THE EDITOR.

* Were this statement correct, Mr. Want feels that he should not only deserve the execration of the public for having practised a gross fraud upon them, but should have betrayed a *pitiable weakness* in attempting to assume the credit of a discovery, in defiance of a public declaration of his want of title to it, before a crowded society of students and practitioners, who could not fail to strip him of his borrowed plumes, and expose him to merited derision.—The answer to Dr. S. and his anonymous informant is in the hands of the printer; but it has been thought adviseable to defer it, on account of the pressure of matter more interesting to the profession than controversial squabbles.

